# Super Bolus: a remedy for a high glycemic index meal in children with type 1 diabetes on insulin pump therapy?—study protocol for a randomized controlled trial

**DOI:** 10.1186/s13063-022-06173-4

**Published:** 2022-03-29

**Authors:** Emilia Kowalczyk, Katarzyna Dżygało, Agnieszka Szypowska

**Affiliations:** 1 Department of Pediatric Diabetology and Pediatrics, Pediatric Teaching Clinical Hospital of the Medical University of Warsaw, Warsaw, Poland; 2 Department of Pediatrics, Medical University of Warsaw, Warsaw, Poland

**Keywords:** Super Bolus, High glycemic index, Postprandial hyperglycemia

## Abstract

**Background:**

Postprandial hyperglycemia (PPH) is a common clinical problem among patients with type 1 diabetes (T1D), which is related to high glycemic index (h-GI) meals. The main problem is linked to high, sharp glycemic spikes following hypoglycemia after h-GI meal consumption. There is a lack of effective and satisfactory solutions for insulin dose adjustment to cover an h-GI meal. The goal of this research was to determine whether a Super Bolus is an effective strategy to prevent PPH and late hypoglycemia after an h-GI meal compared to a Normal Bolus.

**Methods:**

A total of 72 children aged 10–18 years with T1D for at least 1 year and treated with continuous subcutaneous insulin infusion for more than 3 months will be enrolled in a double-blind, randomized, crossover clinical trial. The participants will eat a h-GI breakfast for the two following days and receive a prandial insulin bolus in the form of a Super Bolus 1 day and a Normal Bolus the next day. The glucose level 90 min after the administration of the prandial bolus will be the primary outcome measure. The secondary endpoints will refer to the glucose levels at 30, 60, 120, 150, and 180 min postprandially, the area under the blood glucose curve within 180 min postprandially, peak glucose level and the time to peak glucose level, glycemic rise, the mean amplitude of glycemic excursions, and the number of hypoglycemia episodes.

**Discussion:**

There are still few known clinical studies on this type of bolus. A Super Bolus is defined as a 50% increase in prandial insulin dose compared to the dose calculated based on the individualized patient’s insulin-carbohydrate ratio and a simultaneous suspension of basal insulin for 2 h. Our patients reported the best experience with such a combination. A comprehensive and effective solution to this frequent clinical difficulty of PPH after an h-GI meal has not yet been found. The problem is known and important, and the presented solution is innovative and easy to apply in everyday life.

**Trial registration:**

ClinicalTrials.gov NCT04019821

## Background

Many medical reports, together with our clinical practice, indicate that postprandial hyperglycemia (PPH) is an everyday struggle for people with type 1 diabetes mellitus (T1D), even when metabolic control seems to be adequate based on HbA1c levels. However, the definition of PPH is still not clear or reproducible. The American Diabetes Association (ADA) does not differentiate post-meal norms. The National Institute for Health and Care Excellence (NICE) established post-meal norms at a level above 162 mg/dl (9 mmol/l), whereas the International Society for Pediatric and Adolescent Diabetes (ISPAD) establishes them above 180 mg/dl (10 mmol/l) [1].

The glycemic peak is a common consequence of ingesting carbohydrate-rich meals [2]. To achieve the postprandial glycemic target, carbohydrate (CHO) counting can be a crucial factor [3–6]. A single mealtime insulin dose will cover a range of CHO amounts, with the insulin dose calculated for a meal containing 60 g CHO covering 10 g variations in CHO quantity (50–70 g) [7]. Interestingly, the postprandial glycemic peak rises with increasing CHO intake in a range of 20–80 g of CHOs, but meals containing over 80 g do not cause a greater glycemic peak and instead cause prolonged hyperglycemia [8, 9]. PPH is most often preceded by high glycemic index (h-GI) meals, which causes great glycemic variability, leading to a fast hyperglycemia increase followed by the rapid decline of glucose levels [10–14]. The area under the blood glucose curve (AUC) is 20% larger after the h-GI meal containing the same amount of CHOs compared to a low glycemic index (l-GI) meal [13]. It was also proved that in T1D patients, CHO-based meals caused an increase in the blood glucose level peak within 60–90 min with variations among individuals [10, 11, 15]. PPH and rapid and large glycemic fluctuations are adverse prognostic factors and are related to the development of cardiovascular complications, enhancement of oxidative stress, retinopathy, and certain types of cancers [5, 16]. Furthermore, a correlation between poor glycemic control and negative psychological outcomes, such as depressive symptoms, has been reported in teenagers (10–16 years) [17]. Although it is indicated that patients with T1D should consume l-GI products, the recommendation is rarely followed, especially in the pediatric population [18, 19].

One of the most important goals of T1D treatment is to imitate physiological insulin secretion as closely as possible, thereby maintaining blood glucose levels within the normal range. Previous studies have shown that early preprandial rapid-acting insulin analog administration up to 15–20 min before a meal resulted in lower postprandial glucose excursions compared to 30 min, taken directly at the start of eating [20, 21]. This strategy resulted in a lower rate of PPH without an increased risk of hypoglycemia. Other strategies, such as an additional dose of insulin, were also considered as a possible solution to the h-GI meal issue. Previous studies showed that a 30% increase in the insulin dose led to lower postprandial glycemia and did not cause a higher incidence of hypoglycemia episodes, but the frequency of hyperglycemia remained high [22]. Over the last few years, the idea of “Super Bolus” as a potential solution to the h-GI meal problem has been observed and practiced by some patients every day. This type of bolus is not clearly and unequivocally defined. The general establishment of a bolus is related to the removal of basal insulin and boosting of prandial insulin [23].

The proposed solution of Super Bolus is a combination of two components:

1.An increased dose of prandial insulin (50%) for the quick coverage of h-GI meals

2.Basal insulin was stopped during the following 2–4 h to account for the increased levels of active insulin in circulation after intake of the bolus to prevent hypoglycemia.

There is a lack of clinical studies concerning this type of bolus, and the available literature only refers to in-silico studies and clinical practice [23, 24]. A comprehensive and effective solution to this important clinical problem presented above, which is frequent especially in the pediatric population, has not yet been established. This study aimed to determine whether Super Bolus is more effective than Normal Bolus in preventing PPH and avoiding late hypoglycemia after an h-GI meal in children with T1D treated with continuous subcutaneous insulin infusion.

## Methods

### Trial design

This study was designed as a randomized, double-blind, crossover study with an allocation ratio of 1:1. The trial was registered at ClinicalTrials.gov (NCT04019821) before the inclusion of the first patient. Any important changes in the protocol are introduced.

### Study settings and participants

The participants will be recruited from the Department of Pediatric Diabetology and Pediatrics at the Pediatric Teaching Clinical Hospital, Medical University of Warsaw, Poland. In case of a low recruitment rate, the Diabetic Outpatient Clinic, Pediatric Teaching Clinical Hospital, Medical University of Warsaw, Poland, would also be a reliable source of participants. The hospital is a tertiary referral center that provides medical care for more than 1000 children with T1D. The medical staff are adequately trained and competent in conducting clinical trials. The research will be conducted in accordance with the ethical standards and the Helsinki Declaration of 1964, as revised in 2013. In case of any changes, appropriate information will be added to the protocol registry site, and the bioethics committee will be informed. The principal investigator is responsible for the preparation of the protocol and revisions, the preparation of case report forms (CRFs), data collection and completion of the CRFs, randomization, recruitment of patients, reviewing the progress of the study, and data verification. Research physicians are responsible for data collection and the completion of CRFs, randomization, and recruitment processes.

### Eligibility criteria

Inclusion criteria are children aged 10–18 years with T1D as defined in the ISPAD Guidelines 2018 [25] with a duration longer than 1 year and those who have undergone insulin pump therapy for ≥ 3 months. Patients with celiac disease, diabetes-related complications (e.g., nephropathy), and those that are obese (defined as body mass index [BMI] at or above the 95th percentile for children and teens of the same age and sex) are excluded. The presence of comorbid conditions and treatment, which could significantly affect the glycemic values in the researcher’s opinion, and those who have withdrawn their consent to participate in the study will also be excluded. Written informed consent to participate in the study must be provided by the patients’ parents (and from the patient if they are older than 16 years).

### Intervention

The intervention will involve the administration of insulin for an h-GI breakfast in the form of Super Bolus. The participants will eat a h-GI breakfast for the two following days and receive a prandial insulin bolus in the form of a Super Bolus 1 day and a Normal Bolus the next day. The h-GI breakfast consists of breakfast cereal, cornflakes with added cold milk: 50 g CHO will come from the cornflakes and 10 g of CHO from the 2% milk (200 ml). Super Bolus is defined as the 50% increase in prandial insulin dose compared with the dose calculated based on the individual patient’s insulin-carbohydrate ratio (ICR) and the simultaneous suspension of basal insulin for 2 h. The definition of Super Bolus is based on the patients’ best experiences. Normal Bolus will be defined as the prandial insulin dose calculated based on the individual’s ICR.

### Study procedure

The study procedure is listed in Table 1. Patients who meet the eligibility criteria will be asked to enter the trial during hospitalization. Patients and their parents will receive oral and written information about the study. Verbal consent will be obtained from all participants. If the caregivers agree to participate, written informed consent will be obtained from the legal caregivers and participants older than 16 years by the recruiting physician who is familiar with the study protocol. Prior to the study, the participants will be hospitalized and qualified for a run-in period, which will last approximately 1 week. During this period, the doses of insulin will be adjusted. The 9-point glucose profile will be handled by the nurses, and based on the ICR glucose values and basal insulin rate, it will be optimized by a diabetologist to meet target fasting and postprandial glycemia. After achieving the glucose targets and adjusting the meals according to the ICRs, the allocation process of the participants will be started.

**Table 1:** Timeline of the study

Study period							
	Enrollment	Run-in	Allocation	Post-location			Close-out
Time point	0	0	0	Day1	Day 2	Day3	Day 3
**Enrollment**							
Eligibility screen	x						
Informed consent	x						
Adjusting insulin doses		x					
Allocation			x				
**Interventions**							
A new infusion set				x			
CGM application				x			
Super Bolus, Normal Bolus					x	x	
**Assessments**							
Anthropometric measurement (body weight, height, BMI)			x				
TDD, basal insulin			x				
HbA1c			x				
Blood glucose level 30, 60, 90, 120, 150, and 180 min after prandial bolus					x	x	
Occurrence of hypoglycemia					x	x	
Register records from CGM							x
AUC, peak glucose, time to peak, glycemic rise, MAGE							x

*Abbreviations*: *CGM* Continuous glucose monitoring system, *BMI* Body mass index, *TDD* Total daily dose of insulin, *AUC* Area under the blood glucose curve, *MAGE* Mean amplitude of glycemic excursion

The participants will be randomly divided into two groups: SuBo-NoBo (Super Bolus-Normal Bolus) or NoBo-SuBo (Normal Bolus-Super Bolus). The study will last for three consecutive days. On the first day, a new infusion set with the reservoir and insulin will be inserted to minimize the chance of any leakages and occlusions. Steel needle sets and soft cannulas will be used, depending on the subject’s choice. There will be no restrictions on the insertion site of the set, except for the infected and lipodystrophy areas. The continuous glucose monitoring (CGM) system that will be used will consist of an Enlite™ sensor with a MiniLink™ Transmitter and a MiniMed® Paradigm VEO™ insulin pump (Medtronic MiniMed, Northridge, CA). Proper calibration of the device must be performed. To ease glycemic excursions during the study, participants can take the last correction insulin bolus and any modifications concerning basal insulin 3 h before the meal bolus. To avoid asymptomatic nocturnal hypoglycemic episodes, glycemic control during the nights preceding the test meals will be intensified by performing additional blood glucose meter tests at 12 p.m., 3 a.m., and 5 a.m.

On the second and third days, the test meal (h-GI breakfast) will be given in the morning. The fasting self-monitoring of blood glucose (SMBG) will be performed with a Contour® Plus Link meter with a one-step calibration of CGM. If the fasting glucose value is above 130 mg/dl (> 7.2 mmol/l), the test day will be postponed until the next day. The level was established according to the ISPAD 2018 Guidelines concerning pre-meal targets [1]. Pre-meal insulin will be administered 15 min before the h-GI breakfast as a Super Bolus (group SuBo-NoBo) or Normal Bolus (group NoBo-SuBo) on the second day and the Normal Bolus (group SuBo-NoBo) or Super Bolus (group NoBo-SuBo) on the third day. A 15-min time interval preceding breakfast was established in previous studies as optimal based on the rapid-acting insulin analog onset of action [20, 21]. The participants will use rapid-acting insulin analogs (aspart, lispro, and glargine), as applied thus far. The h-GI breakfast, planned by a clinical dietician, will consist of cornflakes (50 g of CHO) and 2% milk (10 g of CHO), with a total of 60 g of CHO. The test meal contains the following: Nestle Cornflakes with a glycemic index (GI) of 81 and a glycemic load (GL) of 40.5, while 2% ultra-high temperature (UHT) milk has a GI of 27 and a GL of 2.7. The overall GI of the test meal is 72. Despite the low GI of milk, it is a problem that milk products produce high insulinemic indexes of 90–98 [26]. The GI of the food was estimated based on the International Glycemic Index Tables, using bread as a reference. The h-GI test meal (GI 84) consisting of a ham sandwich and a drink used in the study conducted by Ryan et al. resulted in significantly higher postprandial glucose excursions at all time points between 30 and 180 min compared to the low-GI meal (*p <* 0.02). The maximum difference between the postprandial glucose excursions after each test condition occurred at 60 min, and the high-GI meal was 75.6 mg/dl (4.2 mmol/l) higher than that of the low-GI meal (*p <* 0.0001) [14].

The calculation of insulin doses will be performed based on the individual’s ICR counted per 10 g of CHO. The normal bolus will be calculated as 6 × ICR, and the Super Bolus will be calculated as 150% × 6 × ICR, with the suspension of basal insulin for 2 h after the delivered prandial bolus. The CRF containing the protocol for both groups, the personal data, and the calculations concerning insulin doses will be filled in for both groups on the first day. A closed envelope with patient allocation will be attached to the CRF. In the next 2 days, patients will eat h-GI breakfast, and the bolus order will depend on the allocation group. Nurses who will not be involved in the study will administer the right type of bolus. To ensure that the study protocols will not be mixed up and remain blind, the nurse administering the premeal bolus will check the allocation in the envelope and will provide the appropriate type of bolus. The study duration per day is planned for 3 h. No additional meals, snacks, or correction boluses are allowed during this time. An observation time of 3 h is defined as an approximation of the total lasting hypoglycemic effect after a subcutaneous insulin bolus dose. The capillary blood glucose level will be measured with a Contour® Plus Link Meter at 0, 30, 60, 90, 120, 150, and 180 min after insulin administration with a continuously working monitoring system. There is a high possibility that rapid glycemic fluctuations will be observed after an h-GI meal. Therefore, the blood glucose level can be checked with both the CGM and glucometer to obtain the highest accuracy of glycemic level at the specified time point. Caregivers and patients will also be instructed to report symptoms of hypoglycemia with additional glycemia measurements. Hypoglycemia will be defined as the glucose level below or equal to 70 mg/dl (≤ 3.9 mmol/l), which is treated as a clinical hypoglycemia alert according to the ISPAD Guidelines 2018.^1^ If hypoglycemia occurs, the patient will receive 0.3 g/kg of glucose/saccharose. After 15 min, the glucose level must be controlled again. If hypoglycemia persists, the patient will receive the next dose of glucose/saccharose, until a glucose level over 70 mg/dl is achieved. The presence of hypoglycemia will not suspend the study process, and SMBG will be performed as it is established. The records from the CGM will be registered using the Medtronic Care Link Pro Software and discussed with the patients for educational purposes. The participants may discontinue the trial at any point in time without giving a reason. The researchers will make every effort to supervise, educate, and regularly control the patients to provide the proper study process.

### End points

The following is the primary endpoint:

- Capillary blood glucose level 90 min after the administration of the prandial bolus as meals with h-GI typically cause blood glucose level peak within 60–90 min in T1D patients

The following are the secondary endpoints:

- Capillary blood glucose levels at 30, 60, 120, 150, and 180 min after administration of the prandial bolus
- The number of hypoglycemia episodes based on SMBG

The following data are based on the CGM:

- Glycemic rise (GR)—the difference between the baseline and the maximum glucose value
- Peak glucose level (PG)—the maximum value of glycemia during 3 h of post-mealtime
- Time to PG
- Area under the blood glucose curve (AUC)
- Mean amplitude of glycemic excursion (MAGE)—the standard deviation of blood glucose (SDBG) obtained from all blood glucose concentrations within 3 h of post-meal time
- Time in the postprandial glucose range between 70 and 180 mg/dl (4.0–10.0 mmol/l)

### Participant timeline

The study time scheme for enrollment, interventions, and assessments is presented in Table 1.

### Sample size

The sample size was estimated based on the calculations performed using the StatsDirect statistical software (V.3.1.4, StatsDirect, Chesire, UK). A total of 72 participants will be required to show a difference of 30 mg/dl (1.7 mmol//l) and an SD of 41 (which corresponds to the standard deviation of the paired differences being 82) and standard deviations for observations within treatment being 58 at the 90th minute of the study (the primary endpoint), with *α* = 0.05 and 80% power—assuming a 20% withdrawal rate. The correlation was set to 0 (conservative approach). We assume that glycemia differences between the study groups mentioned above are significant for metabolic control.

### Randomization

The participants will be randomly assigned to two groups: SuBo-NoBo or NoBo-SuBo. The randomization list will be generated using the statistical program Stats Direct (V.3.1.4, StatsDirect, Chesire, UK). Blocked randomization (blocks of four) will be used to ensure a good balance of participant characteristics in each group. The randomization list will be kept by a staff member who is not involved in the trial.

### Blinding

All participants and investigators will be blinded to the study procedures. The investigator will be given randomly generated treatment allocations within sealed opaque envelopes. Once a patient has consented to enter the trial, an envelope will be opened, and the allocated treatment regimen will be applied. A nurse not involved in the trial will program the bolus of prandial insulin in compliance with the patient’s allocation found in the sealed envelope and calculated dose of insulin. The screen of the insulin pump will be covered by a piece of black tape to avoid interference. After completing the randomized controlled trial by all subjects, sealed envelopes containing the allocation group of each person will be handed to the principal investigator. The investigator will maintain blindness as much as possible. In cases of exceptional circumstances (e.g., when knowledge of the actual intervention is essential for further management of the patient), the intervention will be unblinded. The principal investigator, in cooperation with research physicians, will decide whether the unblinding procedure is necessary. The reason for unblinding will be reported in the patient’s CRF.

### Data collection and management

A CRF based on the International Conference on Harmonization Guidelines for Good Clinical Practice will be completed on paper for each participant. Data will be transferred to an electronic password-protected database.

The data will be double-entered with automatic checking for mismatches and out-of-range values. All study data documents will be stored in a locker within the study site available only to the staff involved in the research. Data concerning insulin requirements, HbA1c values, and anthropometric parameters will be gathered from non-compliant participants and those who cancel their consent. Only the involved researchers will have access to the dataset and the participant’s personal information. The data will not be shared with any company or founding institutions. They will also not influence the reliable presentation of the results. A draft of the final article with the outcomes will be revised by professional editing services in English. No later than 2 years after the data collection, we will deliver a completely deidentified dataset to an appropriate data archive for sharing purposes.

### Compliance

We will provide written feedback to all the parents and participants about the results of the study when the recruitment process is completed. During the study procedure, a research physician will visit the participant every 30 min (until 180 min), check the blood glucose level, and give a reminder on the rules of the study (i.e., no snacking, no exercising, no delivery of any additional insulin bolus during the observation period).

Compliance with the study protocol will be evaluated by analyzing the information from the CRFs, insulin pump, and recorded CGM data. If participants do not follow the study protocol (i.e., receive an inappropriate dose of insulin, set an incorrect bolus, get an extra insulin dose or snack, or basal insulin suspension is not set appropriately), they will be considered non-compliant. Data from non-compliant participants will be used to perform an intention-to-treat analysis as a sensitivity analysis. If any CGM failure occurs during the study, only data from the glucometer will be analyzed. If the study regimen is interrupted by some factors, data concerning glucose values from the CGM and glucose meter will be collected until that moment.

### Monitoring

The study procedure will be performed according to the manufacturer’s protocol. We do not intend to change the study protocol after recruiting the first patient. If some circumstances that influence the research conditions occur, the changes will be noted at ClinicalTrials.gov (the protocol registry site), and the Bioethics committee will be notified. An independent data and safety monitoring board (DSMB) will be established before the beginning of the study. The DSMB will review the data after the recruitment of 25%, 50%, and 75% of participants to evaluate the study progress and adverse events.

### Statistical analysis

Descriptive statistics will be calculated to characterize and present the study population and baseline findings. Data normality distribution will be verified using the Shapiro-Wilk test. For normally distributed continuous variables, comparative analyses will be performed using Student’s *t-*test. Continuous variables that are not normally distributed will be compared using the Mann-Whitney *U* test. Group comparisons for nominal variables will be conducted using the Fisher exact test or the *χ*^2^ test, as deemed appropriate. Linear regression analysis with the baseline value of the outcome as a covariate will be used to compare the treatment groups. Log transformation will be performed in case of apparent violation of the normal distribution of the data. All tests will be two-tailed, and differences will be considered significant at the level of *p* ≤ 0.05. The number of missing data points will be presented for each variable. Nominal variables will be presented as *n* (% of the group). The outcomes will be presented as differences in medians for continuous data with non-normal distribution and as differences in means for data with normal distribution, both with a 95% confidence interval.

The clinical inference will be based on 95% confidence intervals for the regression coefficient for treatment effect. If the 95% confidence interval does not include 0, we will conclude that the difference between the treatment groups is clinically significant.

Data from the CBGM and CGM will be analyzed separately. To evaluate the capillary blood glucose levels at 30, 60, 90, 120, 150, and 180 min after the administration of the prandial bolus and the number of hypoglycemia episodes, data from the CBGM will be used. Data from CGM will be adopted to calculate the GR, PG, time to PG, MAGE, and time in the postprandial glucose range between 70 and 180 mg/dl (4.0–10.0 mmol/l).

The AUC will be calculated geometrically by applying the trapezoid rule. The incremental area under the blood glucose response curve (iAUC) is defined as the sum of all sensor excursions from the baseline value for the 3-h post-meal period. The positive AUC (pAUC) will be calculated based on the intersection points of the estimated curve with the baseline and integration of the area above the baseline.

Intention-to-treat analysis will be performed as a primary approach. Additionally, the data of participants who will finish the study according to the protocol (per-protocol) will be used as a sensitivity analysis.

### Harms

We do not expect any severe complications during the study. The medical equipment and insulin used during the study will be approved for clinical use. The most probable and harmful adverse effect during the postprandial period is hypoglycemia. Intensive glycemic control using both CGM and SMBG will be used against severe hypoglycemic events and will provide the most accurate glycemic measurements. Despite the inconvenience of having to monitor the blood *glucose levels by doing* multiple finger *pricks, it is necessary to ensure the safety of the study procedure. Moreover, CGM-related minor local adverse events, such as the possibility of developing an infection*, redness, *bleeding*, hypersensitivity, itching, irritation, or pain at the sensor site, may occur. CGM will be applied by qualified personnel to reduce the risk of complications. Data concerning all harms will be gathered and reported as indicated in the Consolidated Standards of Reporting Trials (CONSORT) extension on harms document [27]. Any adverse events will also be reported in the individual patient’s CRFs.

All serious adverse events will be immediately reported to the project leader who will be responsible for notifying the ethics committee and all participating investigators. If any serious adverse events occur during the study procedure, financial compensation will be covered by the grant.

## Discussion

According to the ISPAD Guidelines, low-GI products are recommended for diabetic patients to alleviate glycemic variations [20]. Meals with the same CHO content but different GIs produce clinically significant changes in a person’s postprandial glycemic excursions, as proven in a controlled study among children with T1D that showed that substituting a diet with high-GI foods with a diet comprised of low-GI foods improved glycemic control after 12 months [20].

Some strategies may be implemented to reduce the average GI of meals. The addition of a moderate amount of protein, healthy fats (monounsaturated fatty acids [MUFA] and polyunsaturated fatty acids [PUFA]), or dietary fiber to a meal containing predominantly CHOs may assist in reducing postprandial hyperglycemia [20]. A meal-time schedule, the routine period when food is offered and available without snacking in between meals, may contribute to the optimal control of postprandial glycemia [20].

Adjustment of the type and dose of insulin for h-GI meals remains a challenge. According to Dżygało et al., neither of the two insulin analogs, glulisine nor aspart, provide a stabilized glycemic profile after an h-GI meal [28]. The new formulation of faster-acting insulin aspart (faster aspart) has a more rapid onset of appearance and greater early exposure in comparison with aspart for children and adolescents. Using this new formulation, a statistically significant reduction of 2 h for postprandial glycemia after a standardized meal was reported [29, 30]. However, due to regulatory approval and availability, its common use in clinical practice is limited. Lujif et al. demonstrated that the administration of prandial rapid-acting insulin analogs 15 min (instead of 30 min) before a meal, directly at the start of eating, resulted in a lower rate of PPH without an increased risk of hypoglycemia [21]. Therefore, we decided to deliver an insulin bolus 15 min before a meal. O’Connel et al. tested two types of prandial bolus for an h-GI meal: a normal bolus (given over 3 min before the meal) vs dual (50:50% over 2 h) and found no difference between them [10]. Regardless of the bolus type, high glycemic excursions were still observed, with a mean glycemic rise of + 95.4 mg/dl compared to the baseline [10]. An additional dose of insulin was also considered as a possible solution for the h-GI meals issue. Groele et al. compared the dose of prandial insulin calculated based on the individual patient’s ICR with the dose increased by about 30% for an h-GI meal and concluded that the frequencies of PPH and hypoglycemia were similar in both groups, but the additional dose of insulin significantly reduced glucose excursion in terms of the mean postprandial glycemia (47.4 ± 39.8 mg/dl vs 76.2 ± 58.2 mg/dl) [22]. We know that a dose of insulin increased by 30% does not cause a higher incidence of hypoglycemia episodes and leads to lower postprandial glycemia, but the incidence of hyperglycemia episodes still remains unsatisfactory. Therefore, we decided to conduct this study based on the Super Bolus idea to achieve a reduction in the frequency of PPH, with a simultaneous decrement in glycemic rise and hypoglycemia episodes. The available pertinent literature only refers to in-silico studies and clinical practice [23, 24]. We defined a Super Bolus as a boost of prandial insulin (increased by 50% in comparison with the dose calculated based on individualized patient’s ICR) and a simultaneous suspension of the basal insulin for 2 h. During that time, the insulin is still acting, and 2 h seems to be a short period for a rebound effect. Moreover, our patients reported the best experience with the above-mentioned combination.

A comprehensive and effective solution to this huge and frequent clinical difficulty of PPH after an h-GI meal has not yet been found. The problem is known and important, and the presented solution is innovative and easy to apply in everyday life.

In conclusion, the findings of this randomized controlled aim to show that pediatric patients report the best experience with a combination of a Super Bolus—the 50% increase in prandial insulin dose in comparison with the dose calculated based on the individualized patient’s ICR—and a simultaneous suspension of basal insulin for 2 h. This study will contribute to the formulation of better recommendations on the use of Super Bolus to address problems concerning PPH after an h-HI meal in children with T1D.

### Trial status

Recruitment started in January 2020 and is planned to end in July 2022, with all patients randomized. The current protocol version was 2.0, dated August 30, 2020.

## Data Availability

The full protocol will be freely available because of open-access publication. The findings of this randomized controlled trial will be submitted to a peer-reviewed journal. Abstracts will be submitted to relevant national and international conferences. Standards from the guidelines of the Consolidated Standards of Reporting Trials will be followed for this trial. All investigators will have access to the final trial dataset.

## Abbreviations

PPH: Postprandial hyperglycemia
T1D: Type 1 diabetes
h-GI: High glycemic index
AUC: Area under the blood glucose curve
MAGE: Mean amplitude of glycemic excursion
ICR: Insulin-carbohydrate ratio
CHO: Carbohydrate
SuBo: Super Bolus
NoBo: Normal bolus
CGM: Continuous glucose monitoring system
SMBG: Self-monitoring of blood glucose
GI: Glycemic index
GL: Glycemic load
GR: Glycemic rise
PG: Peak glucose level
SDBG: Standard deviation of blood glucose
CRF: Case report form
DSMB: Data and Safety Monitoring Board
iAUC: Incremental area under the blood glucose curve
pAUC: Positive AUC
CONSORT: Consolidated Standards of Reporting Trials
